# Root exudation in a sloping Moso bamboo forest in relation to fine root biomass and traits

**DOI:** 10.1371/journal.pone.0266131

**Published:** 2022-03-24

**Authors:** Erika Kawakami, Mioko Ataka, Tomonori Kume, Kohei Shimono, Masayoshi Harada, Takuo Hishi, Ayumi Katayama

**Affiliations:** 1 Graduate School of Bioresource and Bioenvironmental Sciences, Kyushu University, Nishiku, Fukuoka, Japan; 2 Research Institute for Sustainable Humanosphere, Uji, Kyoto, Japan; 3 Shiiba Research Forest, Kyushu University, Shiiba, Miyazaki, Japan; 4 Faculty of Agriculture, Kyushu University, Nishiku, Fukuoka, Japan; 5 Kasuya Research Forest, Kyushu University, Sasaguri, Fukuoka, Japan; Government College University Faisalabad, PAKISTAN

## Abstract

Exudation by fine roots generally varies with their morphological traits, but the effect of belowground resource availability on the root exudation via root morphological traits and biomass remains unknown. We aimed to determine the effects of morphological and physiological traits on root exudation rates and to estimate stand-scale exudation (*E*_*stand*_) by measuring the mass, length, and surface area of fine roots in a Moso bamboo forest. We measured root exudation as well as morphological and physiological traits in upper and lower plots on a slope with different belowground resource availability. The mean (± S.D.) root exudation rates per mass in the upper and lower slope were 0.049 ± 0.047 and 0.040 ± 0.059 mg C g^-1^ h^-1^, respectively, which were in the range of exudation found in woody forest ecosystems. We observed significant relationships between root exudation per mass and root respiration, as well as specific root length and surface area. In contrast, exudation per length and area did not correlate with morphological traits. The morphological traits did not differ between slope positions, resulting in no significant difference in root exudation per mass. Fine root biomass, length, and surface area on a unit ground basis were much higher in the lower than those in the upper slope positions. *E*_*stand*_ was higher when estimated by mass than by length and area because the morphological effect on exudation was ignored when scaled using mass. *E*_*stand*_ was 1.4–2.0-fold higher in the lower than that in upper slope positions, suggesting that the scaling parameters of mass, length, and area determined the *E*_*stand*_ estimate more than the exudation rate per mass, length, and area. Regardless of scaling, *E*_*stand*_ was much higher in the Moso bamboo forest than in other forest ecosystems because of a large fine-root biomass.

## Introduction

Plant roots exude soluble organic carbon in the form of amino acids, organic acids, sugars, phenolics, and other secondary metabolites [1]. Root exudation stimulates microbial activity and decomposition in the rhizosphere [2, 3]. Microbial activity and mineralization are more abundant in the rhizosphere, than in bulk soil, and root exudation contributed up to one-third of the total carbon and nitrogen mineralization in temperate forest soils [4]. Thus, root exudation plays an important role in carbon and nutrient cycling in forest ecosystems, and root exudation should be estimated to understand carbon cycling. Despite its importance, few studies have investigated or estimated root exudation in forest ecosystems in situ because of difficulties associated with measurements [5, 6].

The root exudation rate per mass varies with environmental factors such as belowground nutrient [7] and soil water [8] availability, as well as solar radiation [9]. Biotic factors such as mycorrhizal types and life forms [10–12] also affect root exudation rates. These biotic factors can alter root exudation rates by affecting morphological traits such as specific root length (SRL), specific root area (SRA), and root tissue density (RTD) [6, 11, 12]. For example, root exudation rate increases with increasing SRL and SRA and decreasing RTD [13]. Physiological traits, such as root respiration rates, are also important determinants of root exudation rates [6, 14]. The ratios of carbon allocation to exudation and respiration change with soil nitrogen availability [14]. Thus, associations between root exudation rates and morphological and physiological traits are key for understanding the factors that control exudation rates and for estimating stand-scale root exudation in forest ecosystems.

Belowground resource (water and nutrient) availability is an important factor affecting root morphological and physiological traits. For example, fine root respiration, SRL, and RTD are higher in soils with high nitrogen availability [15]. Belowground resource availability can also change fine root biomass. For example, a larger fine root biomass was observed in the upper slope position where nitrogen availability was lower [16, 17]; however, a larger fine root biomass was also observed in the lower slope position where the aboveground net primary production (NPP) and biomass was larger than in the upper slope position [18]. Thus, differences in belowground resource availability might affect fine root morphological traits and fine root biomass, and consequently, stand-scale root exudation rates.

Moso bamboo (*Phyllostachys pubescens*) can reach a height of 20 m, and it has expanded to abandoned farms and forests in East Asia, especially in mountainous areas. The characteristics of carbon cycling in Moso bamboo forests, such as higher NPP [18, 19] and nitrogen storage and absorption, have been investigated [18, 20]. Although root exudation might contribute to NPP in bamboo forests, this has not been investigated. Root exudation rates per mass might differ among locations on slopes according to belowground resource availability, and the variations in root exudation can be explained by root morphological traits and respiration rates. Slope position might also affect fine root biomass, resulting in disparities in stand-scale exudation.

In this study, we aimed to determine the relationship between root exudation and morphological and physiological traits, and to estimate stand-scale root exudation at upper and lower locations of a slope in a Moso bamboo forest with different nitrogen availability in soil [18]. Stand-scale root exudation over a width of 10 m was estimated using fine root biomass, length, and surface area.

## Materials and methods

### Study site

This study proceeded in an abandoned Moso bamboo forest in the Kasuya Research Forest of Kyushu University, Sasaguri, western Japan (33° 37’ N, 130° 32’ E). The mean annual temperature and precipitation were 15.9°C and 1833 mm, respectively, according to a weather station of Kyushu University forest located 3.5 km from the study site [21]. The soil and substrate were brown forest soil and Sangun metamorphic rock, respectively [22], with 6%, 29%, 17%, and 47% clay, silt, sand, (soil < 2 mm) and gravel (soil > 2 mm), respectively [23]. The bulk densities in the upper and lower plot were 0.39 and 0.45 g cm^-3^, respectively [24]. The site is a pure Moso bamboo forest with few understory plants that had never been fertilized, thinned, or cut.

We established 10 × 10 m^2^ plots in the upper and lower slope locations. The distance between the plots was 115 m, and the altitude difference was 79 m. The aboveground biomass and belowground resource availability differed according to the position on the slope [18]. The volumetric soil water content and soil C/N ratios were 24.7% ± 1.7% and 11.3 ± 0.60, and 30.8% ± 2.6% and 13.0 ± 0.19 in the upper and lower plots, respectively. Total soil carbon and nitrogen contents were 4.23% ± 0.62% and 0.38% ± 0.05%, and 3.48% ± 0.25% and 0.27% ± 0.02% in the upper and lower plots, respectively. Aboveground biomass, culm density, and the diameter at breast height (DBH) were 59.4 Mg ha^-1^, 6900 culms ha^-1^, and 7.3 cm in the upper plot, and 166.2 Mg ha^-1^, 9700 culms ha^-1^, and 10.5 cm in the lower plot, respectively.

### Measurements of root exudation

Root exudation was sampled in 16 fine roots per plot during August 2019. The flux rates of root exudation were measured using a syringe-based collection protocol [5] as described [14]. Briefly, living fine roots of one to three branching orders were carefully excavated from the top 5-cm layer of soil, gently washed, and placed in a syringe containing acid-washed glass beads and 25 mL of a carbon-free nutrient solution (0.1 mmol L^-1^ KH_2_PO_4_, 0.2 mmol L^-1^ K_2_SO_4_, 0.2 mmol L^-1^ MgSO_4_, 0.3 mmol L^-1^ CaCl_2_) [5]. The syringes were covered with wet paper towels and aluminum foil to avoid sunlight and minimize heat. The controls comprised two similarly processed syringes without roots from each plot. The nutrient solutions were collected after 24 h of incubation in situ. The syringes were flushed twice more with nutrient solution (10 mL) to fully recover exudation carbon. Solutions were passed through 0.22-μm Minisart^®^ sterile syringe filters (Advantec, Tokyo, Japan) immediately after collection. All syringes were collected and transported on ice to the laboratory. Total organic carbon (TOC) in the root exudation was analyzed using a TOC analyzer (GE Analytical Instruments, Urmston, UK). The root exudation rate was determined by subtracting the mean TOC in the control syringes from each syringe that included roots. Root exudation was calculated based on unit root dry mass per hour (mg C g^-1^ h^-1^), root length (μg C m^-1^ h^-1^), and root surface area (mg C m^-2^ h^-1^). Although 32 fine root exudation were sampled, we determined the root exudation rate of 22 samples because of measurement failure (11 samples in the upper and lower plots, each). The ranges of dry mass, length, and surface area for the sampled roots were 0.045–0.064 g, 0.07–0.62 m, and 0.96–7.10 cm^2^, respectively.

### Measurements of root respiration

Root respiration was measured using a closed static system with a chamber attached to a GMP343 infra-red gas analyzer (Vaisala Oyj., Vantaa, Finland) immediately after root exudation was sampled. The harvested roots were placed in the chamber, and CO_2_ concentrations were measured every second for 10 min using an MI70 portable controller (Vaisala Oyj.). Root respiration rates (F_*root*_, mg C g^−1^ h^−1^) were calculated as:

$$F_{root}=\Delta {CO}_{2}\times \frac{V_{s}}{22.4}\times \frac{273.2}{273.2+T}\times \frac{12}{w}\times 60\times 60\times 10^{-3}$$

where ΔCO_2_ is the CO_2_ concentration increment (ppm) per second, *V*_*s*_ is the chamber volume (0.201 L), the standard gas volume is 22.4 L, the molecular mass of C is 12.0 g mol^-1^, *T* is the mean chamber temperature (T) during measurements, and *w* (g) is the dry mass of the root according to a previous study [2, 14].

### Measurements of root morphological traits

The roots were stored at 4°C after respiration was measured, then scanned at 300 d.p.i. in full color using a Perfection V700 Photo flatbed scanner (Seiko Epson, Tokyo, Japan). Total root length (m), surface area (cm^2^), volume (cm^3^), and mean diameter (mm) were analyzed using a WinRHIZO Pro 2013 image analysis system (Regents Instruments Inc., Quebec City, QC, Canada). The roots were oven-dried at 70°C for 48 h, then weighed. The mean diameter (mm), SRL (m g^-1^), SRA (cm^2^ g^-1^), and RTD (g cm^-3^) were calculated.

### Statistical analysis

The means of fine root exudation, respiration, and morphological traits (diameter, SRL, SRA, and RTD) were compared between the upper and lower plots on the slope using Student t-tests. The effects of root morphological and physiological traits on root exudation were assessed using linear regression analysis with exudation as the objective variable, and the diameter, SRL, SRA, RTD, and respiration rate as the explanatory variables. Root exudation per mass, length, and surface area were used in these analyses. The relationship between root exudation and respiration was compared with that of woody species [14] to understand carbon allocation to exudation and respiration. All data were statistically analyzed using R v3.3.2 (R Foundation for Statistical Computing, Vienna, Austria) with a significance threshold of *P* < 0.05.

### Stand-scale root exudation estimation

Annual stand-scale root exudation (*E*_*stand*_, g C m^-2^ y^-1^) was estimated based on the dry mass, root length, and surface area. The *E*_*stand*_ was estimated on a dry mass basis by multiplying the mean root exudation per root dry mass by the mean root biomass on a ground basis (g m^-2^). The *E*_*stand*_ based on root length was estimated by multiplying mean root exudation per unit length by mean root length on a ground basis. The *E*_*stand*_ based on root surface area was estimated by multiplying the mean root exudation per surface area by the mean root surface area on a ground basis. The fine root biomass, root length, and surface area for the *E*_*stand*_ estimate were measured during December 2018. Soil cores with a diameter of 5 cm and a depth of 10 cm were extracted at five locations in each plot. Fine roots with diameters < 2 mm were separated from the soil, oven-dried at 70°C for 48 h and weighed. The root length and surface area were also analyzed as described above. These values were multiplied by the soil sampling area of the soil cores and calculated on a ground basis.

## Results

### Comparisons of root exudation, respiration, and morphological traits between slopes positions

The range of root exudation rates was 0.002–0.206 mg C g^-1^ h^-1^ with a mean (± S.D.) of 0.045 ± 0.01 mg C g^-1^ h^-1^. The mean root exudation rates per dry mass in the upper and lower plots were 0.049 ± 0.047 and 0.040 ± 0.059 mg C g^-1^ h^-1^, respectively (Table 1). Root exudation rates per root length in the upper and lower plots were 3.72 ± 3.28 and 2.88 ± 3.11 μg C m^-1^ h^-1^, respectively. Root exudation rates per surface area in the upper and lower plots were 3.28 ± 2.44 and 2.44 ± 2.70 mg C m^-2^ h^-1^, respectively. Exudation rates per unit mass, length, and area did not significantly differ between locations on the slope (Table 1). Mean root respiration rates were 0.27 ± 0.14 and 0.32 ± 0.19 mg C g^-1^ h^-1^ in the upper and lower plots, respectively, and did not significantly differ. The diameter, SRL, SRA, and RTD for the sampled fine roots did not significantly differ between the locations of the plots.

**Table 1:** Root exudation, respiration, and morphological traits at upper and lower plots on slope.

Plot	Root exudation			Root respiration (mg C g^-1^ h^-1^)	Diameter (mm)	SRL (m g^-1^)	SRA (cm^2^ g^-1^)	RTD (g cm^-3^)
	per mass (mg C g^-1^ h^-1^)	per length (μg C m^-1^ h^-1^)	per surface area (mg C m^-2^ h^-1^)					
Upper	0.049 ± 0.047	3.72 ± 3.28	3.28 ± 2.44	0.27 ± 0.14	0.35 ± 0.08	15.1 ± 5.5	155.4 ± 33.8	0.77 ± 0.12
Lower	0.040 ± 0.059	2.88 ± 3.11	2.44 ± 2.70	0.32 ± 0.19	0.40 ± 0.10	12.5 ± 5.9	141.7 ± 41.4	0.76 ± 0.11
*P* ^a^	0.67	0.55	0.45	0.51	0.25	0.31	0.40	0.84

Values are shown as mean ± S.D. SRL, specific root length; SRA, specific root area; RTD, root tissue diversity.

^a^Student t tests.

### Relationships of root exudation with morphological traits and respiration

**Fig 1:**
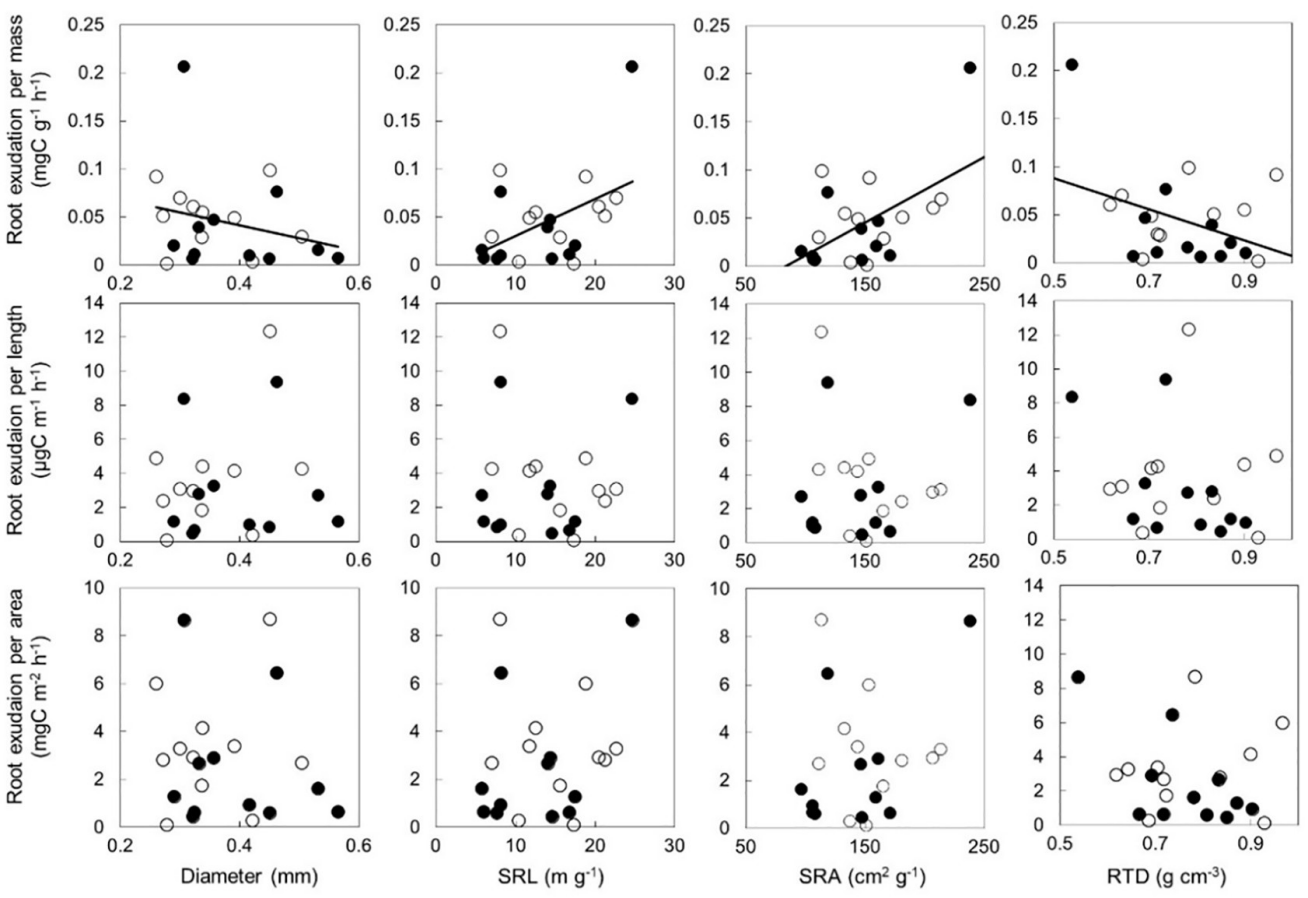
Relationships between diameter, specific root length (SRL), specific root area (SRA), and root tissue density (RTD) with root exudation per mass, length, and surface area. Correlations between exudation per mass and morphological traits were significant, whereas others were not. Unfilled and filled circles indicate upper and lower plots on slope, respectively.

**Fig 2:**
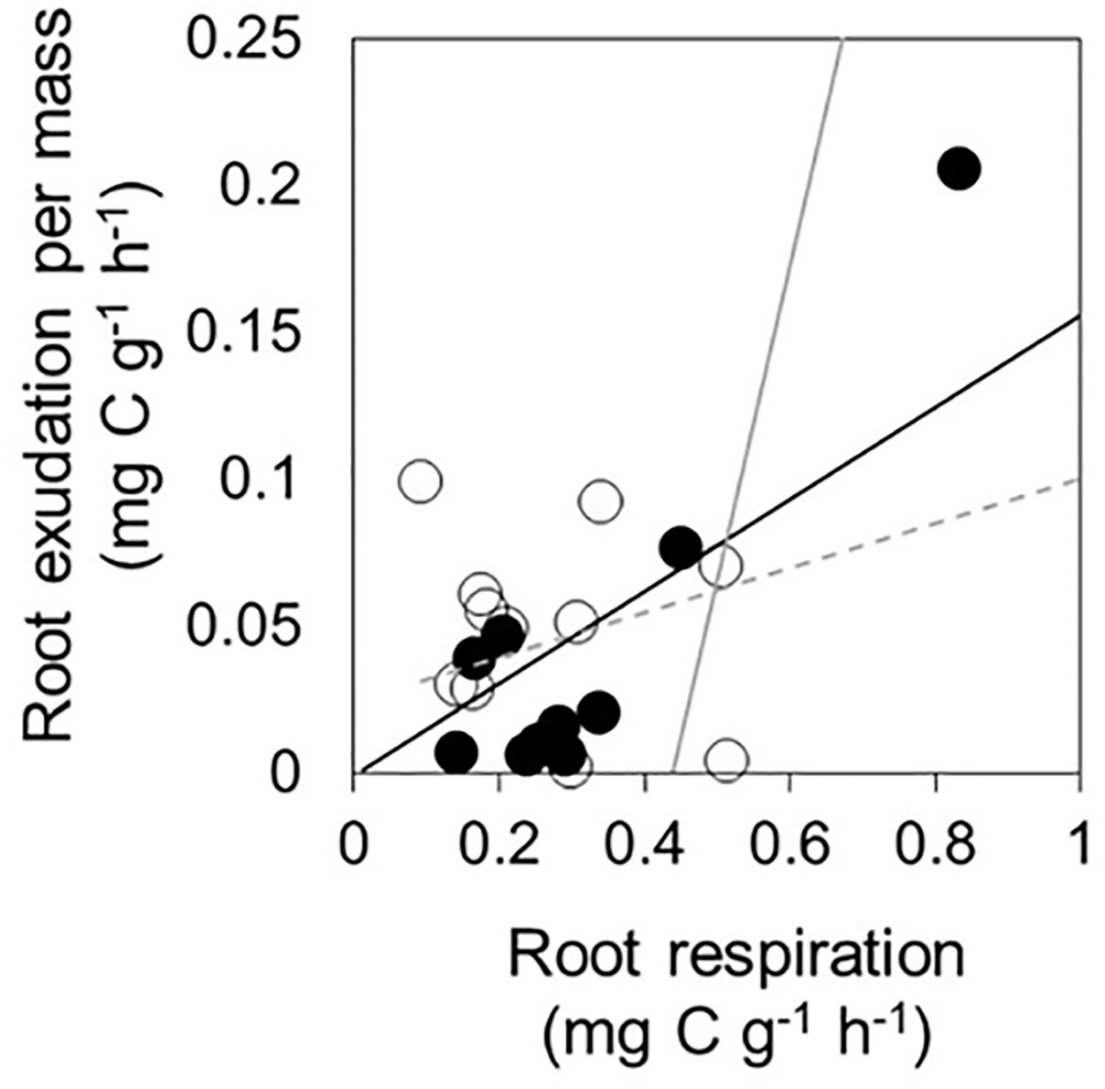
Relationships between root respiration and exudation per mass. Unfilled and filled circles indicate upper and lower plots on slope, respectively. Solid line, correlation between root respiration and exudation (exudation = 0.16 × respiration + 0.00, R2 = 0.31, P < 0.01). Gray and dotted lines, correlation in cool-temperate [14] and temperate [2] Quercus forests, respectively.

Exudation rate per mass significantly correlated with diameter, SRL, and SRA, whereas exudation rates per unit length and surface area did not correlate with any morphological traits (Fig 1). The root exudation rate per mass significantly correlated with root respiration rate (Fig 2).

### Stand-scale root exudation estimate

Root biomass was larger in the lower plot (1078.6 ± 44.9 g m^-2^) than the upper plot (452.3 ± 95.3 g m^-2^). Fine roots were longer and the surface area was larger in the lower plot (7614.3 m m^-2^ and 10.9 m^2^ m^-2^, respectively) compared with the upper plot (4138.5 m m^-2^ and 5.4 m^2^ m^-2^, respectively). The mean diameter of fine roots was 0.42 ± 0.01 and 0.45 ± 0.03 mm in the upper and lower plots, respectively. The *E*_*stand*_ on a mass basis was 2.0-fold higher in the lower plot (382.6 g C m^-2^ y^-1^) compared with the upper plot (194.8 g C m^-2^ y^-1^; Table 2). The *E*_*stand*_ on a length basis was the smallest among the estimates and was 1.4-fold higher in the lower plot (192.4 g C m^-2^ y^-1^) than in the upper plot (134.7 g C m^-2^ y^-1^). The *E*_*stand*_ on an area basis was 1.5-fold higher in the lower plot (233.2 g C m^-2^ y^-1^) than in the upper plot (156.1 g C m^-2^ y^-1^). The ratio of root exudation to fine root production estimated by Shimono et al. [18] in this study site (218 and 248 g C m^-2^ y^-1^ in the upper and lower plots, respectively) varied according to the estimation method and ranged between 0.62 and 1.54 (Table 2).

**Table 2:** Stand-scale root exudation (*E*_*stand*_) estimated by root dry mass, root length, and root surface area.

Plot	Per mass				Per length				Per surface area			
	Exudation (mg C g^-1^ h^-1^)	Mass (g m^-2^)	*E*_*stand*_ (g C m^-2^ y^-1^)	Ratio to FRP	Exudation (μg C m^-1^ h^-1^)	Length (m m^-2^)	*E*_*stand*_ (g C m^-2^ y^-1^)	Ratio to FRP	Exudation (mg C m^-2^ h^-1^)	Area (m^2^ m^-2^)	*E*_*stand*_ (g C m^-2^ y^-1^)	Ratio to FRP
Upper	0.049	452.3	194.8	0.89	3.72	4138.5	134.7	0.62	3.28	5.4	156.1	0.72
Lower	0.040	1078.6	382.6	1.54	2.88	7614.3	192.4	0.78	2.44	10.9	233.2	0.94

Fine root production (FRP, g C m^2^ y^-1^) was estimated using the plot by Shimono et al. [18].

## Discussion

### Effects of slope position on root exudation

Root exudation rates per unit mass, length, and surface area did not significantly differ between the upper and lower slopes (Table 1). Although SRL and SRA significantly correlated with root exudation rates per unit mass, these morphological traits for the sampled fine roots did not differ between the upper and lower plots, resulting in no significant difference in root exudation rate per mass (Table 1). A previous study in the same bamboo forest reported a higher belowground NPP allocation and lower nitrogen use efficiency in the upper plot than in the lower plot, suggesting lower water availability in the upper plot [18]. Despite the difference, we did not find significant differences in morphological traits for the sampled fine roots. These results imply that the difference in belowground resource availabilities between slope positions was not large enough to identify significant differences in SRL and SRA, and therefore, root exudation rate per mass. On the other hand, values for fine root biomass, length, and surface area on a ground basis were higher in the upper than the lower plot, resulting in a higher *E*_*stand*_ in the lower plot. These results indicate that fine root biomass determined *E*_*stand*_ than root exudation rate per mass, length, or area.

### Relationships of exudation with morphological traits and respiration

Exudation rates per mass and morphological traits were significantly related (Fig 1) and were consistent with previous findings [6, 13]. Root exudation positively correlates with competitive traits such as SRA on an individual scale in 18 tree species [6]. Our results showed that competitive fine roots with higher SRA or SRL and higher exudation was observed for at least one species under natural conditions. Exudation and respiration also correlated significantly and positively (Fig 2). The relationship is the same in forest ecosystems [2, 14], whereas exudation did not increase with respiration in response to temperature or belowground carbon allocation in wheat [25]. The authors explained that root exudation is a passive phenomenon caused by the difference in concentration between root tissue and soil, whereas root respiration is necessary for tissue production and maintenance, which results in a different response between respiration and exudation. Nevertheless, our results show that exudation consumed 16% of the carbon used for respiration in this bamboo forest, which was lower than the 49–111% and 44% identified in a cool-temperate [14] and a temperate [2] *Quercus* forest. These findings indicate that allocation to exudation in the C flux is lower for Moso bamboo roots than for woody species.

### Stand-scale exudation estimate

We found that *E*_*stand*_ was dependent on root mass, length, and surface area (Table 2). The relationship between the exudation rate per mass and SRL as well as SRA was positive (Fig 1). Therefore, the exudation rate per mass depending on these two parameters should be measured to improve the accuracy of *E*_*stand*_ estimates. That is, the average exudation rate per mass when used for scaling ignored the effects of morphological traits, resulting in *E*_*stand*_ overestimation. Accordingly, the value of *E*_*stand*_ estimated using mass was higher than that using either length or area, suggesting that exudations from thicker roots can be overestimated using mean exudation based on relatively thin roots. On the other hand, exudation determined based on length and area did not differ with any morphological traits, suggesting a constant exudation per unit length or area. Thus, root length and surface area might be more suitable as scaling parameters for *E*_*stand*_ estimates rather than mass.

We found several ambiguities associated with the stand estimate. The fine roots used to estimate *E*_*stand*_ comprised all roots with a diameter < 2 mm, whereas those used to measure exudation included only those of one to three branching orders. Accordingly, diameter of the fine roots for exudation measurement (0.35 and 0.40 mm in the upper and lower plots, respectively) was thinner than fine roots for biomass measurement (0.42 and 0.45 mm in the upper and lower plots, respectively). Whether the relationship between exudation and SRA or SRL can be applied to fine roots of more than three branching orders is unknown. Thinner roots generally with a low branching order are physiologically more active than thicker roots [26, 27]. Thus, the exudation of thicker fine roots should be evaluated to increase the accuracy of *E*_*stand*_ estimates. In addition, we considered fine root biomass only at a depth of 0–10 cm. Fine root biomass at a depth of 0–30 cm was 2222 and 1071 g m^-2^ in the lower and upper plots, respectively [18], which was twice as high as that at 0–10 cm. Whether fine roots in deeper soil release comparable amounts of exudation should be assessed to improve the accuracy of *E*_*stand*_ estimates. Furthermore, temporal and seasonal variation in exudation should also be considered for the annual estimate, although the annual *E*_*stand*_ in the present study did not consider temporal variation. Previous studies examined temporal variation in exudation rate, but the results were contradictory. For example, solar radiation affected root exudation for *Quercus* trees, whereas seasons did not affect exudation rate [9]. No significant differences in exudation for pine trees were also found among dates and seasons [5]. In contrast, exudation was higher in a young Chinese-fir forest during summer than that during winter [28]. Future studies examining temporal and seasonal variation in root exudation for different tree species are necessary.

### Exudation in the Moso bamboo forest

This study is the first to describe root exudation in a bamboo forest. The root exudation rate of Gramineae grass is 0–1.0 mg C g^-1^ h^-1^ [29]. Root exudations of broadleaved trees measured in the manner described herein are 0.016–0.065 mg C g^-1^ h^-1^ for beech trees [12], 0.073 ± 0.011 mg C g^-1^ h^-1^ for *Quercus* trees [2] and 0.004–0.021 mg C g^-1^ h^-1^ for broadleaved trees [11]. The mean exudation in the present study (0.045 ± 0.01 mg C g^-1^ h^-1^) was comparable to these values, suggesting that Moso bamboo and woody species release comparable amounts of carbon as root exudations per mass. On the other hand, *E*_*stand*_ values in the present study (134.7–382.6 g C m^-2^ y^-1^) was much higher than those reported in previous studies. For example, the annual *E*_*stand*_ was estimated at 4–16 g C m^-2^ y^-1^ in beech forests because of low fine root biomass (53–110 g m^-2^), which was much lower than those in the present study (1078.6 ± 44.9 and 452 ± 95.3 g m^-2^ in the lower and upper plots, respectively). The lower estimate was also caused by temporal scaling, that is, the annual *E*_*stand*_ was estimated by multiplying daily exudation rates by the average length of the growing season (186 days). The *E*_*stand*_ in *Quercus* forests was estimated at 15 g C m^-2^ y^-1^, which was calculated based on the relationship of exudation rate with root respiration and annual root respiration data [2]. Root respiration differed with temperature, and the effect of temperature was therefore included in the annual *E*_*stand*_ estimate. The *E*_*stand*_ in tropical rainforests was estimated at 0.21–0.70 mol C m^-2^ mo^-1^, which was equivalent to 30.2–100.8 g C m^-2^ y^-1^ [30]. As they considered only organic acid exudation, the total *E*_*stand*_ might be closer to our estimate. Although our findings of root exudations per mass were comparable to those in woody species, the fine root biomass in the Moso bamboo was far larger than the average in temperate forests (480 ± 261 g m^-2^, whole rooting depth), as calculated from 71 previous studies [31]. The higher fine root biomass was one of the reasons of higher *E*_*stand*_ in the bamboo forest.

The NPP is higher in Moso bamboo forests than in forest ecosystems [19, 20] and in this particular forest [18] because of higher fine root production. Our results imply that the actual belowground NPP in Moso bamboo forests with consideration of root exudation might be considerably higher than previous estimates that excluded root exudation. The high allocation to belowground can be attributed to a high demand for nitrogen [18, 20] and water [23]. Higher nutrient and water absorption abilities can support high carbon assimilation in bamboo forests.

## Supporting information

S1 Table Data measured in this study. (XLSX)
